# A Case Report of Acute Lymphoblastic Leukemia Complicated by Naso-Ophthalmic Mucormycosis

**DOI:** 10.7759/cureus.79984

**Published:** 2025-03-03

**Authors:** Shaohua Yang, Wanli Du, Yu Zhang, Yujie Fan, Ning Wang

**Affiliations:** 1 Ophthalmology, Gansu Provincial Maternity and Child-Care Hospital, Lanzhou, CHN

**Keywords:** acute lymphoblastic leukemia, amphotericin b, fungal infection, nasocerebral mucormycosis, posaconazole

## Abstract

Rhinocerebral mucormycosis, an invasive fungal infection caused by *Mucorales* species, typically involves the paranasal sinuses, orbits, and central nervous system. Known for its aggressive nature, the infection often leads to severe complications and a high risk of mortality. Common risk factors include uncontrolled diabetes mellitus, immunosuppression, prolonged use of glucocorticoids, and hematologic malignancies. Patients with hematologic malignancies are particularly susceptible to this infection due to compromised immune function. This case report details an instance of acute lymphoblastic leukemia complicated by naso-ocular encephalomycosis, providing a comprehensive overview of its clinical presentation, diagnostic approach, therapeutic management, and prognosis. Additionally, preventive measures and early diagnosis are important in patients with hematologic malignancies.

## Introduction

Rhinocerebral mucormycosis (ROCM), a rapidly progressing fungal infection caused by *Mucorales* species, poses a significant threat to immunocompromised individuals, including those with hematologic malignancies like acute lymphoblastic leukemia (ALL) [1,2]. The infection typically begins in the nasal and sinus regions, potentially spreading to the orbits and brain, leading to severe clinical manifestations such as sinusitis, vision impairment, and ocular muscle paralysis [3]. These symptoms often overlap with those of orbital cellulitis or orbital apex syndrome, complicating early diagnosis and necessitating a high index of suspicion [4].

In ALL patients, the risk of mucormycosis is exacerbated by chemotherapy-induced neutropenia and the frequent use of corticosteroids, which further suppress immune function [5]. Effective management requires a multidisciplinary approach, including prompt antifungal therapy with agents like amphotericin B, surgical debridement of necrotic tissue, and correction of underlying predisposing factors such as hyperglycemia or immunosuppression [6]. Despite these measures, the prognosis remains poor, particularly in cases where diagnosis and treatment are delayed [7]. This case report highlights the challenges of managing naso-ophthalmic mucormycosis in an ALL patient, emphasizing the importance of early recognition and tailored therapeutic strategies.

## Case presentation

**Figure 1:**
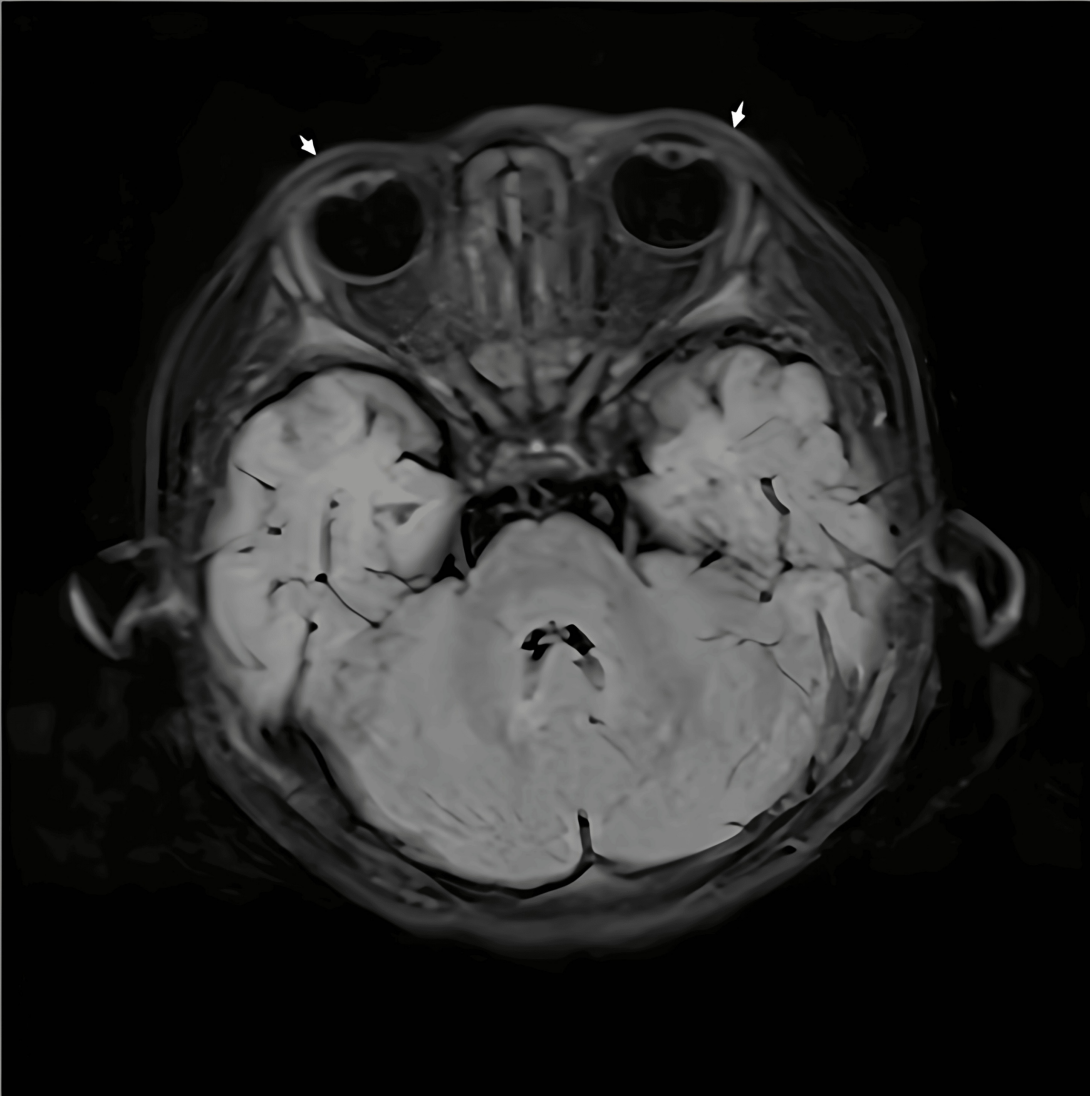
MRI of the head and orbits at the time of admission The figure presents an orbital MRI utilizing T2-weighted FLAIR imaging. The horizontal scan demonstrated the presence of bilateral, localized edema of the eyelids (white arrows). MRI: magnetic resonance imaging, FLAIR: fluid-attenuated inversion recovery

A one-year-old male patient was admitted to the hospital on August 14, 2022, presenting with a five-day history of fever and three days of eyelid edema. Upon physical examination at the time of admission, the patient appeared to be in satisfactory condition, although significant pharyngeal congestion was noted. There were no signs of purulent secretions or herpes lesions, and conjunctival congestion was absent. Auscultation of the lungs revealed coarse breathing sounds, but no dry or wet rales were detected. The abdominal and neurological examinations did not reveal any notable abnormalities. Throughout the course of the illness, the child maintained a clear mental state, although his appetite and sleep patterns were reported to be average, and there was a noted decrease in urine output. The patient's medical history was unremarkable. Laboratory results indicated a white blood cell count of 2.3 x 10^9/L, a C-reactive protein level of 109.98 mg/L, and a platelet count of 81 x 10^9/L. An advanced imaging study, specifically an MRI of the head and orbits, revealed no definitive abnormalities in the brain parenchyma. However, bilateral localized edema of the eyelids was observed (Figure 1). Bone marrow aspiration was performed on a patient diagnosed with ALL. The chemotherapy regimen included intrathecal administration of dexamethasone, methotrexate, and cytarabine, along with intravenous infusion of daunorubicin hydrochloride at a dosage of 15 mg and vincristine sulfate at 0.75 mg.

The patient experienced recurrent fever throughout the hospitalization period, with temperature fluctuations ranging from 37.4 to 39.0°C. The patient was treated with a combination of meropenem and vancomycin. However, no significant clinical improvement was noted following treatment with mycin and fluconazole. On August 20, 2022, cerebrospinal fluid and blood cultures were conducted; the fungal culture did not yield any fungal growth, and the aerobic blood culture remained negative after five days.

A physical examination on September 1, 2022, revealed slight edema at the nasal root, with dark red soft tissue observed at the tip of the nose. By September 3, 2022, the examination indicated increased edema at the nasal root, dark red skin, soft tissue changes in the nose, and involvement of the left inner canthus and middle forehead, with a progressive deterioration of the local lesions. On September 4, 2022, the next-generation sequencing (NGS) of blood samples indicated an infection caused by *Mucor umbellifera*. Consequently, antifungal treatment was initiated, comprising a combination of amphotericin B and posaconazole. On September 5, 2022, an ocular examination revealed significant findings in the left eye. There was elevated orbital pressure upon palpation, and the eyeball exhibited increased firmness. The skin temperature of the eyelid was elevated, accompanied by mild erythema and swelling. Notably, approximately one-third of the inner canthus of the eyelid displayed necrosis, with visible blood exudation at the palpebral margin. The eyeball was protruding, and there was pronounced bulboconjunctival edema. The cornea appeared grayish-white and opaque, with a pupil measuring approximately 4 mm and lacking light reflex. In contrast, the right eyelid showed no signs of congestion or edema, the cornea remained transparent, and the conjunctival eyelid was non-congested. The pupil on the right side demonstrated a normal light reflex and was crystal clear. Fundoscopic examination revealed that the optic disc boundaries in both eyes were well-defined, exhibiting a red hue, with normal blood vessels. There were no significant signs of hemorrhage or exudation in the retina, and the macular light reflex was clear. Ocular B ultrasonography revealed no significant abnormalities. However, partial eyelid necrosis and potential orbital tissue infection are considered. In the otolaryngology assessment, notable findings included swollen ecchymosis surrounding the nasal dorsum and orbit and scattered skin necrosis observed in the turbinate and eyebrow regions.

A chest CT scan conducted on September 5, 2022, revealed bronchopneumonia with lobular consolidation in the upper lobe of the left lung, showing an increase in new lesions compared to previous imaging, while the remaining areas of exudation appeared reduced. Additionally, anemia was noted. The scan also identified a diffuse decrease in liver density, a new finding compared to prior assessments. A physical examination performed on September 6, 2022, revealed edema at the nasal root, localized skin necrosis in the central forehead region, partial skin necrosis of the left eyelid, and stiffening and necrosis of the skin on both sides of the nasal wings, as well as the nasal dorsum and root. Additionally, there was an absence of airflow in the nasal cavity, elevated pressure in the left eye upon palpation of the orbital surface, hardening of the eyeballs, increased skin temperature of the eyelids, blood exudation from the palpebral margins, protrusion of the eyeballs, significant bulbar conjunctival edema, and obscured fundus details.

**Figure 2:**
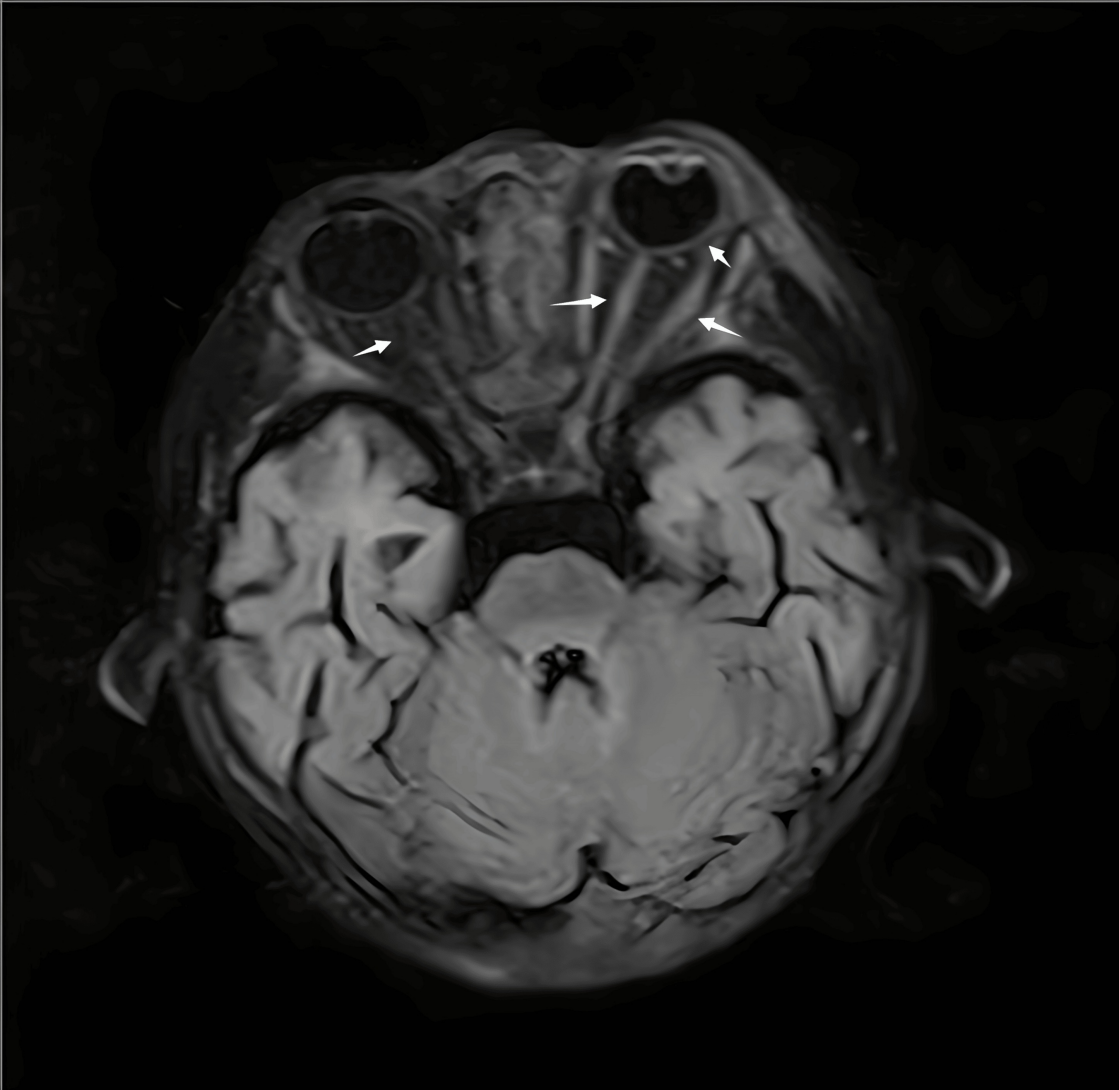
Orbital nuclear MRI on September 6, 2022 This figure presents an orbital MRI utilizing T2-weighted FLAIR imaging. The horizontal scans revealed thickening and increased signal intensity in the eye ring, extraocular muscles, and optic nerve (white arrows). MRI: magnetic resonance imaging, FLAIR: fluid-attenuated inversion recovery

On September 6, 2022, an orbital MRI revealed protrusion of the left eyeball and thickening of the eye ring and optic nerve. Additionally, bilateral eyelid, periocular, and nasal subcutaneous soft tissue edema were observed, which may indicate the presence of infectious lesions (Figure 2). Following a multidisciplinary consultation, it was recommended that debridement of the necrotic tissue be performed. The family members were apprised of the patient's condition and the potential prognosis. Ultimately, the family opted to forgo further treatment. The discharge diagnosis included ALL, cerebral fungal infection, sepsis, hypoproteinemia, pulmonary infection, electrolyte imbalance, and hypokalemia. Later, we followed up with the child's family by phone but could not contact them.

## Discussion

ROCM is a vascularly invasive fungal infection that primarily impacts the sinuses, orbits, and brain. Consequently, initial diagnoses may occur in the ocular region, necessitating a heightened suspicion among clinicians regarding the potential for ROCM. This concern is particularly pertinent for patients exhibiting poor glycemic control or those who are immunocompromised due to diabetes. Over the past two years, there has been a notable increase in ROCM cases globally, with a significant rise observed in India. Research indicates that glucocorticoid use and diabetes are the predominant predisposing factors associated with the development of COVID-19-related ROCM [8]. Therefore, it is imperative that patients recovering from COVID-19 receive regular follow-up care to identify symptoms and signs promptly, as timely diagnosis and early intervention are critical for effective treatment. Statistical data reveal that the average age of ROCM is 54.9 years, with a higher incidence observed in males, who account for 77.4% of cases [9]. The underlying mechanisms contributing to the increased susceptibility of males remain inadequately understood. Additional risk factors for ROCM encompass hematological malignancies, prior organ transplantation, acquired immune deficiency syndrome, intravenous drug use, alterations in iron metabolism, blood transfusions, trauma, and malnutrition [1].

The primary infection route involves inhaling sporangium spores from the environment, which are lodged in the turbinate, sinuses, and alveoli. This can lead to allergic sinusitis and interstitial pneumonia in individuals with a competent immune response. At the same time, in immunocompromised patients, it may result in invasive infections of the sinuses, orbit, and lungs [4]. The principal pathophysiological characteristics of mucormycosis infection include vascular invasion, thrombosis, and tissue necrosis.

The clinical manifestations of ROCM encompass a range of symptoms, including necrosis of the turbinates characterized by a black appearance, bloody nasal discharge, unilateral pain, and swelling in the periorbital or perinasal regions, which may present with discoloration or sclerosis. Additional ocular symptoms may include ptosis, exophthalmos, optic neuropathy, and varying degrees of ophthalmoplegia [3]. The ocular manifestations of ROCM bear a resemblance to those seen in orbital cellulitis and orbital tip syndrome, both of which are complications associated with ROCM and necessitate differentiation. Systemic symptoms may manifest as headache, epistaxis, fever, nasal obstruction, rhinorrhea, and oral pathologies [2]. In the case presented, the initial symptoms included fever, eyelid edema, and edema at the nasal root. As the condition progressed, approximately one-third of the eyelid canthus of the left eye exhibited blackened skin, with blood exudation noted at the palpebral margin. Additionally, there was significant protrusion of the eyeball, pronounced bulboconjunctival edema, a grayish-white and cloudy cornea, a pupil measuring approximately 4 mm with absent light reflex, and no significant abnormalities detected in the fundus. Swelling and ecchymosis were noted in the area surrounding the nasal dorsal orbit, accompanied by localized skin necrosis in the turbinate and eyebrow regions. A nasopharyngeal CT scan conducted on the second day revealed the presence of a brain infection, sinusitis, and infections within the intraorbital and nasal cavities. These findings suggest that the disease associated with rhinosinusitis and orbital involvement (ROM) advances swiftly and exhibits significant invasiveness. Timely detection, accurate diagnosis, and prompt treatment are crucial in preventing the dissemination of ROM to the orbital and cerebral tissues, thereby enhancing the likelihood of successful outcomes.

The management of ROCM necessitates a multidisciplinary approach that includes the prompt administration of antifungal therapy, specifically amphotericin B, alongside the debridement of necrotic tissue. The efficacy of treatment is influenced by several factors, including the level of immunosuppression, the location and severity of the infection, the timeliness of intervention, and the treatment modalities employed [10]. Addressing the underlying condition and mitigating predisposing factors are critical to effective management. For instance, patients with diabetes should maintain optimal blood glucose levels. At the same time, those with hematological malignancies may need to adjust or temporarily discontinue glucocorticoids or immunosuppressants until the mucormycosis is adequately controlled. Amphotericin B is recognized as the primary therapeutic agent for mucormycosis, with its effectiveness substantiated by clinical trials and studies [11]. Furthermore, hyperbaric oxygen therapy may be utilized as an adjunctive treatment for ROCM, potentially enhancing antifungal activity by promoting oxygen free radical production. Recent reports indicate that the combination of hyperbaric oxygen therapy and amphotericin B demonstrates a favorable impact on the treatment of mucormycosis affecting the sinuses, skin, and soft tissues [12]. Nevertheless, there is a notable deficiency of clinical randomized controlled trials that assess its precise efficacy. Managing this infection solely with antifungal agents proves challenging; therefore, early surgical intervention is essential for debridement and removing all necrotic tissue.

## Conclusions

Given the rapid progression of ROCM, clinicians must achieve an early diagnosis and promptly initiate antifungal therapy and surgical debridement to optimize infection control. The management of such cases requires a multidisciplinary approach involving hematologists, infectious disease specialists, and surgeons to address the complex interplay between the underlying malignancy and the aggressive fungal infection. Early recognition of subtle clinical signs, such as sinusitis or orbital swelling, can significantly improve outcomes by enabling timely intervention. Furthermore, this case underscores the importance of close monitoring and tailored treatment strategies for high-risk patients, particularly those with prolonged immunosuppression due to chemotherapy or corticosteroid use. By sharing this experience, we aim to raise awareness of the diagnostic and therapeutic challenges associated with ROCM in ALL patients and to encourage further research into optimizing management protocols.
